# Unauthorized Food Manipulation as a Criminal Offense: Food Authenticity, Legal Frameworks, Analytical Tools and Cases

**DOI:** 10.3390/foods10112570

**Published:** 2021-10-25

**Authors:** Karlo Jurica, Irena Brčić Karačonji, Dario Lasić, Danijela Bursać Kovačević, Predrag Putnik

**Affiliations:** 1 Special Security Operations Directorate, Ministry of the Interior, Ulica Grada Vukovara 33, 10000 Zagreb, Croatia; juricakarlo@gmail.com; 2 Institute for Medical Research and Occupational Health, Ksaverska Cesta 2, 10000 Zagreb, Croatia; ibrcic@imi.hr; 3 Faculty of Health Studies, University of Rijeka, Viktora Cara Emina 5, 51000 Rijeka, Croatia; 4 Andrija Štampar Teaching Institute for Public Health, Mirogojska 16, 10000 Zagreb, Croatia; dario.lasic@stampar.hr; 5 Faculty of Food Technology and Biotechnology, University of Zagreb, Pierottijeva 6, 10000 Zagreb, Croatia; dbursac@pbf.hr; 6 Department of Food Technology, University North, Trg dr. Žarka Dolinara 1, 48000 Koprivnica, Croatia

**Keywords:** food authenticity, criminal offense, unauthorized food manipulation, food defence, mitigation strategy

## Abstract

Food fraud is a criminal intent motivated by economic gain to adulterate or misrepresent food ingredients and packaging. The development of a reliable food supply system is at great risk under globalization, but Food Business Operators (FBOs) have a legal obligation to implement and maintain food traceability and quality at all stages of food production, processing, and distribution. Incidents of food fraud have a strong negative impact on consumer confidence in the food industry. Therefore, local and international regulatory mechanisms are established to prevent or mitigate food fraud. This review brings new perspectives linking EU and US legislation, as well as new definitions and descriptions of the criminal aspect of food fraud incidents. It also describes certain new insights into the application of state-of-the-art methods and techniques that provide valuable tools for geographic, botanical, or other chemical markers of food authenticity. The review also provides an overview of the most common cases of food fraud worldwide from 2010 to 2020. Further research is needed to support the development of predictive models for innovative approaches to adulteration, especially when some valuable nutrients are replaced by toxic ingredients. A possible solution to minimize food fraud incidents is to increase the level of risk-based inspections, establish more productive monitoring and implementation of food protection systems in the supply chain, and implement better ingredient control and certification. National and international (e.g., regional) police offices for food fraud should be introduced, possessing knowledge and skills in food, food safety, food processing, and food products, as initial positive results have emerged in some countries.

## 1. Introduction

Even in ancient times, food traders sought to make greater profits by manipulating the quality of food, disguising the origin of the product, or concealing the expiration date for consumption. Our everyday life is stocked full of numerous cases of adulteration, counterfeiting, and dilution, as well as substitution of ingredients in ready-to-eat dietary products. According to general food legislation, a food is defined in Reg. 2002/178/EC as: “*any substance or product whether processed, partially processed or unprocessed, intended to be or reasonably expected to be ingested by humans*” [1].

**Figure 1:**
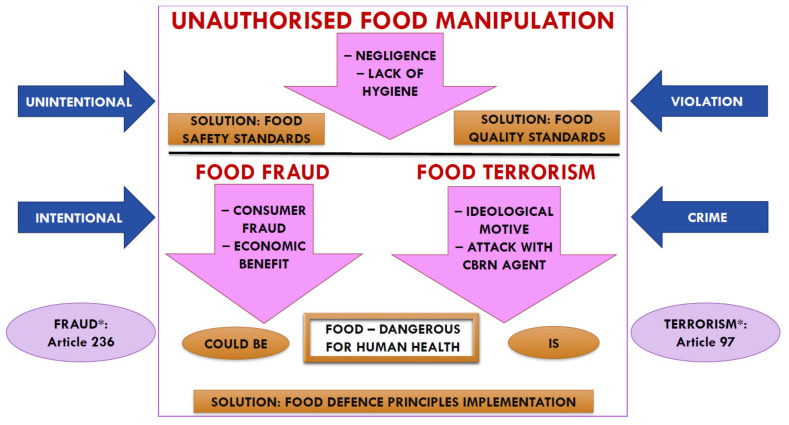
Unauthorized food manipulations (UFMs), law violations, and criminal offenses. * As defined by Croatian Criminal Law (NN 125/11, 144/12, 56/15, 61/15, 101/17, 118/18, 126/19, 84/21).

Food business operators (FBOs) have a legal obligation to comply with food hygiene requirements, and to establish and maintain traceability and quality (of food for human and animal consumption, as well as ingredients used in food) at all stages of production, processing, and distribution. They are also responsible for developing a reliable food supply system, with the ultimate goal of providing consumers with a safe food product. The implementation and certification of basic food safety standards (HACCP, ISO 22000), as well as extended standards (FSSC 22000, IFS, BRC) at FBOs, makes this possible. Therefore, the food safety approach should protect the food supply chain from unintentional contamination, while food defence protects the food supply chain from intentional adulteration that can cause harm [2], as well as from unauthorized food manipulation (UFM) (Figure 1).

A crime is an illegal act or offense punishable by law (i.e., unlawful; contrary to law; act directly prohibited by law). Food crime is an activity with the motivation to deceive or to harm consumers, organized by individuals or groups [3,4]. Spink et al. [5] defined food crime as any type of food fraud that is conducted on a large scale and with serious potential repercussions for public safety or with a significant financial loss for consumers or businesses.

Any criminal activity that may occur during food production, processing, retailing, or distribution can be broadly referred to as UFM. It is a type of crime primarily because it is committed with clear intent and motives. Common motives for committing fraud include making economic profits from a particular food product by using (i.e., substituting with) cheaper ingredients or by tampering with the food product in a way that alters its usual characteristics (Figure 1). If the committed food fraud causes human casualties, it falls under another type of criminal offense (homicide/murder) or more criminal offenses combined into one act (e.g., fraud, production and marketing a food product harmful to human health, endangering of life and property by a generally dangerous act or medium, and murder/homicide). Unfortunately, many incidents of food fraud go unnoticed, because the perpetrators’ main goal is to gain financial benefit and not to cause a health hazard [6].

The European Union (EU) has the criterion that some cases could be fraud if certain conditions are met (as defined in Section 2.1., third paragraph).

Regarding food fraud in the United States (US), the Food and Drug Administration (FDA) has legally defined food adulteration and mislabelling (misbranding) as criminal offenses [7]. The terms food fraud and economically motivated adulteration (EMA) are synonyms according to the available literature [1,8]. Some US authors have subdivided EMAs into individual acts that include adulteration, tampering, theft, diversion, simulation, counterfeiting, and misleading information. However, regardless of the definitions used, both sides point to the same problem, which is fraud against consumers to gain an economic advantage. Croatian national criminal legislation (there is no European general criminal law) defines certain general terms such as fraud, processing and marketing of food harmful to human health, or endangering life and property by generally dangerous acts or media [9].

Nowadays, fraudulent approaches have become more sophisticated and target the molecular level. Therefore, in order to determine the interference in a food product, it is necessary to use analytical forensic methods, which are currently undergoing extensive development. There are already many standardized and mostly specialized analytical laboratory methods, some of which are in the rapid-development phase, with the main objective of detecting the fraudulent activities and providing correct answers. However, analytical methods to date do not give complete solutions for all food fraud activities.

As incidents up to 2010 have been well documented and described in a review paper by Everstine et al. [8], this article aims to provide an overview of the legal framework and terms for the most common cases and methods of food fraud detection between 2010 and 2020 for the EU and USA.

## 2. Legal Framework

In Europe, according to Reg. 2002/178/EC, food also includes beverages, chewing gums, and any substance, including water, intentionally added to food during its manufacturing, preparation, or treatment [1].

An authentic food product is a food that has been produced or grown in such a way that it has a characteristic feature (e.g., nutritional value, sensory properties, specific composition, health-promoting properties) and/or has been produced under certain conditions (e.g., traditional, technologically advanced, or under intellectual property rights). Food authenticity is displayed on the original product packaging and labelled with a text or trademark. Food authenticity refers to product quality and characteristics which depend on food processing and environmental factors (e.g., geolocation, climate, soil type) [10]. Authenticity provides a higher economic value of the product, as it is equal to the additional value of a product.

### 2.1. Definitions and Legislation of Food Fraud in the EU

As already mentioned, it is generally accepted in the EU that food fraud includes cases where EU food law violations exist, and the act is committed intentionally in order to gain economic or financial advantage by deceiving consumers. Food fraud became an important object of interest in 2013, when the horsemeat scandal first emerged [11].

The EU Commission has described food fraud in Reg. 178/2002/EC [1], which provides a specific legal framework to protect consumers. Indeed, Article 8 aims to prevent food fraud or deceptive practices, food adulteration, and other practices that may mislead the consumer. In addition, Article 18 of this Regulation provides information on how to present and label food and feed correctly and not misleadingly.

For an incident to be classified as food fraud in the EU, it must meet the following four criteria [11]. Firstly, there must be a breach of one or more regulations related to food and feed safety legislation. Secondly, there must be an intention to substitute high-quality ingredients with low-quality ingredients (for large quantities of products). This must be verified against numerous factors that provide strong evidence that certain non-compatibilities are not incidental. In fact, some possible impurities in production, when ingredients are substituted for non-essential ingredients, could indicate an intent to deceive (i.e., imply adulteration). Thirdly, the existence of significant economic profits that follow from the addition of non-compatibilities to the product must be established. Fourthly, there must be an element of deception of the consumer, which is the final criterion linking all the other factors to food fraud. This implies that some form of deception, such as color or label changes, has concealed the true quality of the product (or, in worst-case scenarios, even the hazardous nature of the food). For instance, the element of food fraud can often imply concern for public health by hiding some actual harmful product characteristics (e.g., allergenicity, toxicity).

At the member state level, food safety and food fraud were addressed by Reg. 852/2004/EC [12] for food hygiene, while Reg. 178/2002/EC [1] introduced the concepts of crisis management and the obligation to adopt a General Food Crisis Management Plan with the European Food Safety Authority (EFSA). Reg. 178/2002/EC, Article 14 [1] prohibits the placing on the market of unsafe or harmful food, or anything that is unsuitable for human consumption (taking into account additives and pesticides at concentrations above maximum residue levels (MRLs)) according to Reg. 396/2005/EC [13]. 

The Reg. 510/2006/EC serves the protection of geographical origin and designations of origin of agricultural products and foodstuffs [14]. Many foods and beverages in the EU are protected by geographical origin schemes, such as protected geographical indications (PGI), where at least one of the stages of production, processing, or preparation must take place in a specific region, and protected designations of origin (PDO), where all steps must take place in the same specific region [15].

The European Food Fraud Network (EU FFN) was established in response to the horsemeat scandal, and it helps EU countries to operate in accordance with Reg. 625/2017/EC regarding official control. The Directorate General for Health and Food Safety (DG SANTE)—Unit G5 is responsible for communication regarding food fraud incidents.

### 2.2. Definitions and Legislation of Economically Motivated Adulteration in the US

The US FDA defines EMAs as the false, intentional substitution or addition of compounds to increase apparent product value or reduce production costs, and includes food, supplements, tobacco, cosmetics, medications, medical devices, and equipment [16]. EMA in the food industry can occur through manipulation within the original food composition, ingredient substitution or disapproved food processing, and mislabelling of food. Food fraud can include misrepresentation, theft, diversion, simulation, smuggling, counterfeiting, and anything that can be classified as forgery, as described by the FDA, so it is not just the addition or substitution of raw materials [17]. Most commonly, criminal manipulation with food occurs in the form of adulteration and misbranding (mislabelling), as defined in Sections 342 and 343 of the Federal Food, Drug, and Cosmetic Act [16]. 

According to the US literature, economically motivated food adulteration (e.g., adulteration of food or introduction of other materials and ingredients) is most similar to the EU concept of food fraud, with both sides emphasizing the intent to commit a food crime. Moreover, economically motivated food fraud or adulteration is much more common than intentional contamination of food/water. For example, there were 200 reported cases of economically motivated food adulteration in the US between 1980 and 2010 [18]. Such EMA incidents can cause serious harm to public health and are certainly a serious challenge to all of the parties involved, such as the food industry, inspection agencies, and the legal system, as these acts are committed in a manner intended to avoid or minimize food fraud detection [8]. Food Safety Systems (USA) and the current Quality Assurance Methodology (QA) are not designed to detect new non-authentic ingredient adulterants or low levels of non-toxic diluents, and funding for such inspections is limited, even though they pose a serious threat to food systems [8].

## 3. Types of UFM

Responsibility for food adulteration and other food crimes lies with FBOs and the governmental administration [19,20], and internal quality control systems as well as legal monitoring systems are relied upon. In research conducted in Croatia and Serbia, the three main groups of food fraud types were mislabelling (35.8%), adulteration (18.9%), and substitution (13.2%) [19]. 

**Figure 2:**
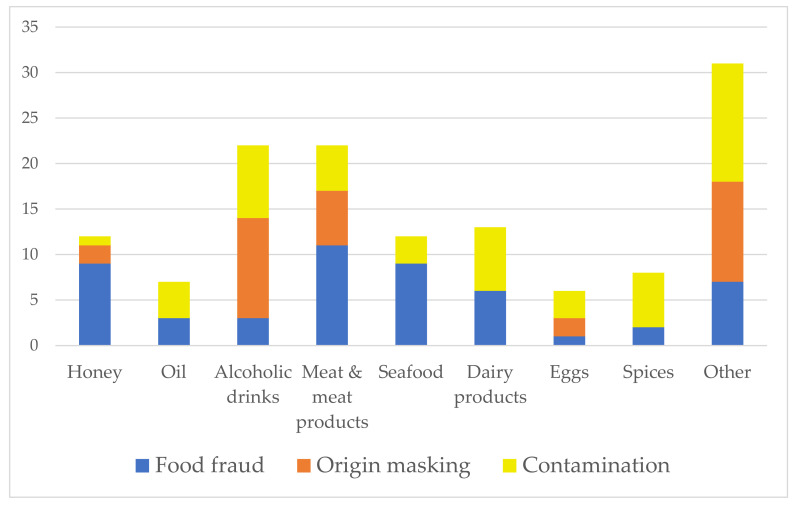
Food products that are most often subjected to unauthorized food manipulation (UFM) (2010–2020). Food fraud—mislabelling, deceiving practices, and ingredients replacement. Origin masking—food authenticity changed (counterfeiting). Contamination—harmful adulterant incorporated into food.

**Figure 3:**
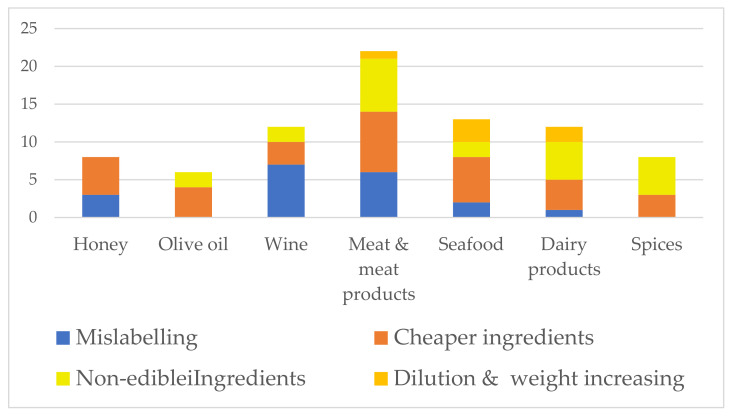
Types of unauthorized food manipulation (UFM) that occur most often in particular food groups (2010–2020).

Looking at the overall state of food, the most frequent adulterations are done in powders and liquids (e.g., olive oils, alcoholic beverages, fish, seasonings, sauces, cereals, and flours) and much less in whole and fresh foods. In terms of food type, meat and meat products, fish and fish products, milk and dairy products, fruits, juices, and honey are the most susceptible to fraud [19,21]. Table 1, Table 2 and Table 3 give a detailed overview of the UFM regarding food fraud cases identified from 2010 to 2020. A summary of the tables is given in Figure 2 and Figure 3.

Adulteration of meat and seafood products involves substitution with species of low commercial value. Although the motivation is mainly economic, it can also affect health; for instance, when meat with low allergenic potential is substituted with meat products with high allergenic potential by adding, for example, soy, gluten, or milk proteins [22,23]. Common frauds also include increasing product weight by adding excessive water to frozen products, changing the stated country of origin, and using illegal chemicals in production [8]. Adulteration of dairy products involves addition of water (e.g., to increase the volume of milk), starch, vegetable oils, and wheat flour to increase the thickness and viscosity of milk. Chemicals such as calcium hydroxide, sodium hydroxide, or formalin are used to increase shelf life, while detergents are used to maintain the frothy appearance that decreases when the milk is diluted with water. Urea is added to impart whiteness and falsely increase nitrogen content, or in other words, to achieve a similar effect to melamine [22,24]. Olive oil is often adulterated by the addition of other oils of lesser commercial value, which usually do not have the same functional properties as olive oil (e.g., vegetable oil). It is even not uncommon to use oils that are not intended for human consumption, such as lampante olive oil [22]. Cheap wines and alcoholic beverages are often labelled as more expensive. While labelling a cheaper wine as more expensive does not pose a risk to human health, alcoholic beverages that may contain methanol are still highly toxic [8]. The main frauds in coffee production are the addition of barley, corn, or rice, or substitution with cheaper coffee of lower quality [23]. Spices often contain toxic adulterants that threaten public health; for instance, spices are colored with lead chromate, azo dyes, and triphenylmethane dyes [22]. Honey is usually adulterated with cheaper sweeteners, such as high-fructose corn syrup, corn sugar syrup, invert sugar syrup, cane sugar syrup, rice syrup, and so on. Additional substitutions may also include cheap low-quality honeys [25].

As noted above, UFM as a legal term describes a broader concept of food fraud that encompasses all possible fraudulent offenses: adulteration and tampering/mislabelling (e.g., green-colored sunflower oil labelled as extra virgin olive oil, sale of flower honey labelled as acacia honey), substitution (maple syrup diluted with cheaper table syrup (sugar), low-quality wine blended with high-quality wine), dilution (diluting milk with water), and deceitful practices (beef substituted for horse meat) (Table 1). The term UFM is used to clarify and define all types of fraudulent acts committed. Fraudulent acts related to the false presentation of food products with PDO (e.g., other grape varieties used for the production of Aceto di Modena) or PGI (e.g., inferior wine labelled as PGI wine from Tuscany) are also part of the UFM (Table 2). Similarly, sometimes impurities are used as adulterants that can cause serious health problems due to their toxicity (e.g., rice wine contaminated by methanol) or allergenic properties (a hazelnut product contaminated with peanuts), as shown in Table 3.

**Table 1:** Unauthorized food manipulation regarding food fraud cases, 2010–2020.

Country, Year	Food	Unauthorized Food Manipulation *		Adulterants	Case	Reference
		Fraud Type (USA), FDA 401, 403	Fraud Type (EU), 178/02 Article 8			
**Honey**						
USA, 2011	Honey	Adulteration Tampering	Mislabelling Deceiving practice Ingredients replacement	Chinese honey	Fifteen people and six companies from all around the world were accused of masking Chinese-origin honey with new packaging and false documents before shipping it to the U.S. for consumption in various forms ^1^.	[26]
Italy, 2017	Acacia honey	Adulteration Substitution/Tampering	Mislabelling Deceiving practice Ingredients replacement	Flower honey	A distributor of local and foreign honeys (Romania, Croatia, and Argentina) was selling flower honey labelled as acacia honey that was 40% more expensive. Twenty-two tons of honey were seized after discovering the fraud using pollen analysis ^1^.	[27]
Australia, 2018	Honey	Adulteration Dilution/ Substitution Tampering	Mislabelling Deceiving practice Ingredients replacement	Corn syrup	An Australian honey distributer was accused of selling honey adulterated with corn syrup to increase profit ^1^.	[28]
South Africa, 2018	Natural honey	Adulteration Dilution Substitution Tampering	Mislabelling Deceiving practice Ingredients replacement	Solution of sugar and lemon	A honey producer from South Africa was accused of preparing concentrated sugar solution spiked with lemon and selling it as honey. The producer denied the accusation but confessed feeding bees with sugar. Products labelled as natural honey were recalled from the stores ^1^.	[28]
Canada, 2019	Imported honey	Adulteration Dilution/Substitution Tampering	Mislabelling Deceiving practice Ingredients replacement	Sugar cane and rice syrup	Tests carried out by the Canadian Food Inspection Agency indicated that 22% of tested imported honey was diluted with sugar cane or rice syrup. Unlike imported honey, none of the Canadian honey tested was adulterated ^1^.	[29]
Italy, 2020	Honey	Tampering	Mislabelling Deceiving practice	Honey without origin label	Seven tons of honey were seized because they lacked a label of origin. This was the same honey that was seized before because of noncompliance with legal hygienic requirements but was placed again on the market illegally ^1^.	[30]
**Olive oil**						
Greece, 2017	Olive oil	Adulteration Substitution Tampering	Mislabelling, Deceiving practice	Sunflower oil dyed green	Sunflower oil dyed green labelled as extra virgin olive oil was sold throughout Europe ^1^.	[27]
Italy, 2019	Olive oil	Tampering	Mislabelling Deceiving practice Ingredients replacement	Chlorophyll, soya oil, beta-carotene	To obtain the right color, chlorophyll, soya oil, and beta-carotene were added to olive oil and sold at ten times greater price ^1^.	[31]
Brazil, 2020	Extra virgin olive oil	Adulteration Tampering Counterfeiting	Mislabelling Deceiving practice Ingredients replacement	Soybean oil	Nine companies sold soybean oil as extra virgin olive oil.	[32]
**Wine**						
France, 2012	High quality wine	Tampering Substitution/ Dilution	Mislabelling Deceiving practice	Low quality wine	The winery was blending low-quality wine with other wines and sold the blends as a high-quality Bordeaux wine to supermarkets ^1^.	[33]
Italy, 2019	Wine	Adulteration Tampering	Mislabelling Deceiving practice	Sugar	In order to increase the alcohol content, the wine company was accused for adding sugar to wine. Around 450,000 litres of wine and 7000 kg of sugar were seized ^1^.	[34]
Spain, 2020	Brandy	Adulteration Tampering Counterfeiting	Mislabelling Deceiving practice	Corn syrup	The Spanish authorities arrested six people that produced adulterated wine. They utilized isoglucose (e.g., corn syrup) to produce wine and alcoholic drinks such as brandy.	[35]
**Meat**						
Europe, 2013	Beef	Adulteration, Substitution	Deceiving practice	Horse meat	Horse meat scandal in 2013 affected all EU member states and 15 other countries. It was discovered that the products being labelled as containing beef (row meat and various prepared meat products such as lasagne, spaghetti Bolognese, chili con carne, moussaka, etc.) were substituted with cheaper horse meat ^1^.	[36]
China, 2014	Donkey meat	Substitution	Deceiving practice	Fox meat	Donkey meat sold at some outlets in China was recalled after tests showed the products contained fox meat ^1^.	[37]
Italy, 2017	Wild boar and deer meat sausages	Substitution	Deceiving practice	Pig meat sausages	Two companies in Puglia were selling wild boar and deer meat sausages actually made from pig meat to gain profit ^1^.	[38]
Mexico, 2017	Ground beef	Adulteration Substitution	Deceiving practice	Ground horsemeat	DNA testing carried out by the School of Veterinary Medicine of Mexico City revealed that 10% of ground beef products contained horsemeat. The majority of vendors claimed that they were not aware of this practice ^1^.	[27]
Spain, 2017	Beef burgers	Adulteration, Substitution	Deceiving practice	Pig meat, soy and bread	A frozen products company has been accused of adding pig meat, soy, and bread to their beef burgers and meatballs. The fraud was discovered by a dismissed employee ^1^.	[38]
UK, 2017, 2018	Chicken meat	Adulteration In UK	Deceiving practice in UK	Chicken meat contaminated with Salmonella	Some 1400 tons of chicken meat infested with Salmonella and originating from Brazil was stopped at the UK border and shipped back to Brazil, where it was later sold as processed meat. The practice is allowed in Brazil because the heat treatment applied during the processing of the meat kills the bacteria. The operation took place between April 2017 and November 2018 ^1^.	[29]
Spain, 2018	Ham	Adulteration Tampering	Mislabelling Deceiving practice	Ham with expired date	More than 10,000 hams with expired dates were discovered in rented containers. The hams were relabelled to extend their expiry date and to resell ^1,2^.	[39]
UK, 2018	Lamb	Substitution	Deceiving practice	Beef	A restaurant was selling grilled beef as lamb. The fraud was discovered when a Trading Standards Officer who bought the meat sent it for analysis ^1^.	[40]
UK, 2018	Lamb	Substitution	Deceiving practice	Mutton	In an Indian restaurant, the inspectors found that lamb dishes (animal < 12 months old) were actually made from mutton (older animal) ^1^.	[41]
France, 2019	Chicken meat	Adulteration Dilution	Deceiving practice	Water	In order to increase the weight, water was added to chicken meat imported from Denmark ^1^.	[42]
France, 2019	Meat burgers	Adulteration Substitution	Deceiving practice	Fat, skin, starch, and soya	Polish burgers (7 million pieces) sold in France contained fat, skin, starch, and soya, which are not authorized ingredients for this type of product ^1^.	[43]
**Sea food**						
Europe, 2017	Fresh tuna	Substitution	Deceiving practice	Canned tuna	Tuna was sold as fresh when it should be sold as canned tuna. Tuna can be sold as fresh only if frozen at −18 °C immediately after being caught and kept at that temperature until arrival at destination. Tuna stored in salt water at −9 °C should be canned. Fresh tuna is three times more expensive than canned ^1^.	[44]
Italy, 2017	Red tuna Grouper	Substitution	Deceiving practice	Yellowfin tuna Nile perch	A well-known hotel suspected of selling low-quality fish species instead of the higher-quality fishes: red tuna was replaced by yellowfin tuna and grouper was replaced by Nile perch ^1^.	[38]
Canada, 2018	Fish	Substitution Mislabelling	Mislabelling Deceiving practice	Other fish species	Authorities in Canada reported that more than 40% of fish samples were replaced with cheaper ones, such as tilapia or Japanese amberjack, which can trigger health effects. More mislabelling of fish species was found in restaurants (55%) than in retailers (22%) ^1^.	[39]
China, 2018	Xue Yu fish	Substitution	Deceiving practice	Other fish species	DNA testing showed that 58% of Chinese premium fish sold as Xue Yu (Mandarin for “Cod”, Gadidae family) belonged to other fish species ^1^.	[45]
Italy, 2018	Wild caught fish	Substitution Tampering Mislabelling	Mislabelling Deceiving practice	Farmed fish	One hundred kilos of sea bass farmed in Greece was intended to be sold at a much higher price to restaurants and fish markets as wild-caught in the Mediterranean ^1^.	[46]
USA, 2018	Atlantic blue crab	Substitution Mislabelling	Mislabelling Deceiving practice	Crab	Due to decreases in catches of the genuine Atlantic blue crab that could not meet consumers’ demand, a food processing company sold crab from Asia, and Central and South America labelled as more expensive Atlantic blue crab ^1^.	[47]
USA, 2018	High quality fish	Substitution/Tampering Mislabelling	Mislabelling Deceiving practice	Low quality fish	In New York supermarkets, more than 85% of high-quality and expensive fish species were mislabelled. The most mislabelled species included lemon sole, red snapper, and “wild” salmon ^1^.	[48]
USA, 2018	Octopus	Substitution Mislabelling	Mislabelling Deceiving practice	Squid	Food processing and distribution companies in Long Island have been selling cheaper squid falsely labelled as expensive octopus ^1^.	[47]
Mexico, 2020	Fish	Adulteration Substitution/Dilution/Tampering Mislabelling	Mislabelling Deceiving practice	Glazed fish	To prevent dehydration of the surface, a thin layer of ice can be added on frozen fish (glazing). In case of glazing, water content in the fish can be up to 57% (versus 30% of average water content in frozen fish without glazing). Glazed fish sold at retailers has no labels indicating glazing ^1^.	[30]
**Dairy products**						
Canada, 2017	Kosher cheese	Mislabelling	Mislabelling Deceiving practice	Non-kosher cheese	A company from Toronto falsified certificates and sold fake kosher cheese to Jewish summer camps in 2015 ^1^.	[38]
Switzerland, 2017	Milk	Adulteration Dilution Tampering	Mislabelling Deceiving practice	Water	A Swiss farmer was accused of diluting milk with water. The dairy company which bought the milk sued the farmer who made a profit of 41,000 EUR and asked for compensation of 120,000 EUR ^1^.	[49]
Africa, 2018	Milk powder	Dilution All types of fraud	Deceiving practice	No animal proteins	Milk powder without animal protein is a common problem in African countries such as Tanzania, Nigeria, Kenya, and Ghana. It has also been stated that 50% of imported goods in Tanzania, including food, are fake ^1^.	[50]
Colombia, 2019	Milk	Adulteration Substitution	Mislabelling Deceiving practice	Whey	Milk adulterated with whey was sold daily in Colombia ^1^.	[51]
Italia, 2019	Cheese	Tampering/Mislabelling	Mislabelling, Deceiving practice	Expired date	Three tons of cheese were seized due to expired date and storing under adverse conditions ^1,2^.	[31]
USA, 2019	Vanilla ice cream	Substitution/Mislabelling Tampering	Mislabelling Deceiving practice	Ingredients other than vanilla	An ice cream producing company was accused of selling ice cream labelled as vanilla ice cream that contained neither vanilla nor vanilla extract. The vanilla flavor was likely obtained from ingredients other than vanilla ^1^.	[52]
**Spices**						
Denmark, 2017	Oregano	Adulteration Dilution	Mislabelling Deceiving practice	Ground dry leaves from other plants	In 4 out of 10 samples tested by the consumer association, pure oregano contained ground dry leaves from olive or myrtle trees ^1^.	[53]
UK, 2019	Saffron	Adulteration Substitution	Mislabelling Deceiving practice	Fibers from other plants	The Food Standards Agency detected fibres from other plants in saffron originating from Spain. As a consequence, almost 90 kg of saffron was seized in a factory in Alicante ^1^.	[29]
**Other**						
Italy, 2018	Tomato juice	Tampering Mislabelling	Mislabelling Deceiving practice	Expired date	More than 200 tons of tomato juice was removed from the market when it was discovered that the producer falsely prolonged the shelf life of jarred tomato juice by replacing the expiry date label ^1,2^.	[47]
Italy, 2018	Frozen food	Tampering Mislabelling	Mislabelling Deceiving practice	Expired date	Sixteen tonnes of frozen foods have been seized after discovering that the expiry date was relabelled for selling despite having expired several years ago ^1^.	[39]
Canada, 2018	Maple syrup	Adulteration Tampering Dilution	Mislabelling Deceiving practice	Table (sugar) syrup	USA customs discovered that maple syrup from Quebec was diluted with cheaper table (sugar) syrup ^1^.	[45]
Portugal, 2020	Eggs	Tampering, Mislabelling	Mislabelling Deceiving practice	Expired date	Almost 50,000 eggs have been relabelled to extend their expiry date to increase the durability of the product ^1,2,3^.	[54]
Italia, 2019	Pesto sauce	Tampering/Mislabelling	Mislabelling Deceiving practice	Frozen ingredients	Six hundred kg of pesto sauce was mislabelled to mask the origin and quality. The pesto was made with frozen ingredients ^1,2^.	[31]
Africa, 2018	Rice	Dilution All types of fraud	Deceiving practice	Rice husk	Rice husk sold as high-quality rice is a common problem in African countries such as Tanzania, Nigeria, Kenya, and Ghana. It has also been stated that 50% of imported goods in Tanzania, including food, are fake ^1^.	[50]
Brazil, 2019	High quality rice	Substitution Adulteration	Mislabelling Deceiving practice	Low quality rice	In 2018, more than 40% of the high-quality rice controlled on the Brazilian market was mixed with lower quality rice ^1^.	[34]
India, 2020	Protein powder	Adulteration Tampering Counterfeiting	Mislabelling Deceiving practice Ingredients replacement	Steroids	A plant was manufacturing hundreds of kilograms of fake protein powder, mislabelled as produced by top USA or EU companies. The supplements contained banned steroids.	[32]

* Unauthorized Food Manipulation (types of criminal offenses) according to Croatian Criminal Law (NN 125/11, 144/12, 56/15, 61/15, 101/17, 118/18, 126/19, 84/21): ^1^ Article 236: Fraud; ^2^ Article 188: Production and marketing of a product harmful to human health; ^3^ Article 215: Endangering the life and property with generally dangerous act or medium [9].

**Table 2:** Unauthorized Food Manipulation regarding origin masking, 2010–2020.

Country, Year	Food	Unauthorized Food Manipulation *		Adulterants	Case	Reference
		Fraud Type (USA), FDA 401, 403	Fraud Type (EU), 178/02 article 8			
**Honey**						
France, 2018	Corsican PDO honey	Adulteration, Substitution Mislabelling Misleading information (according to Manning & Soon (2014))	Mislabelling Deceiving practice	Chestnut honey	A honey producer from Corsica mixed 600 kg of imported chestnut honey with its own honey produced in Corsica and sold it as “AOP miel de Corse”, which is, beside the “Miel de sapin des Vosges”, the only PDO (protected designation of origins) honey in France ^1^.	[28]
New Zealand, 2019	Manuka honey	Adulteration, Substitution Mislabelling Misleading information (according to Manning & Soon (2014))	Mislabelling Deceiving practice	Methylglyoxal and dihydroxyacetone	A honey producer was adding methylglyoxal and dihydroxyacetone to 14 tons of honey during its processing to imitate premium-quality manuka honey, in which both compounds are naturally present ^1^.	[43]
**Wine, vinegar, and alcoholic drinks**						
Trinidadian rum, 2016	Trinidadian rum	Substitution Counterfeit Mislabelling	Mislabelling Deceiving practice	Cuban and South American rum	A bulk rum from Cuba and South America was falsely labelled as “100% Trinidadian rum” and sold to export markets ^1^.	[55]
Canada, 2018	“Irish cream”	Substitution Counterfeit Mislabelling Misleading information (according to Manning & Soon (2014))	Mislabelling Deceiving practice	Cream liquors	Cream liquors produced in Canada are frequently sold as “Irish cream”, genuine Irish cream liquor with PGI label ^1^.	[48]
Italy, 2019	PGI wine	Adulteration Substitution Counterfeit	Mislabelling Deceiving practice	Lower quality wine	Lower-quality wine labelled as PGI wine from Tuscany (11,000 bottles) was seized by the Italian authorities ^1^.	[34]
Italy, 2019	Verdicchio dei Castelli di Jesi wines	Adulteration Substitution Counterfeit	Mislabelling Deceiving practice	Low quality wine	A total of 15,000 litres of wine was falsely labelled as Verdicchio dei Castelli di Jesi. The fraud was discovered because of its low market prices ^1^.	[31]
Italy, 2019	Grapes for Aceto di Modena	Substitution, Counterfeiting Mislabelling	Mislabelling Deceiving practice	Other types of grapes	Italian authorities have seized 9000 tons of crushed grapes which did not fulfil requirements for producing Aceto di Modena. This PDO product can only be produced with seven grape varieties sourced in certain areas of Italy. The fraud was probably a consequence of the low grape production rate in Italy in 2018 ^1^.	[56]
New Zeeland, 2019	Waipara and Marlborough Sauvignon Blanc vintage wines	Adulteration Substitution Counterfeit	Mislabelling Deceiving practice	Low quality wine	A winery provided incorrect label on geographical origin and vintage for tens of thousands of bottles of wine. The fraud was related to 2011, 2012, and 2013 Waipara and Marlborough Sauvignon Blanc vintages ^1^.	[29]
Spain, 2019	Wine	Substitution, Counterfeit Tampering (according to Spink & Moyer (2011))	Mislabelling Deceiving practice	Low quality wine	A wine company was selling low- to medium-quality wine under the label El Bierzo, a PDO wine from the Spanish province of León. The wine was sold all over the world ^1^.	[31]
Italy, 2020	Organic wine	Substitution Tampering Mislabelling	Mislabelling Deceiving practice	Low quality wine	More than 10 million litres of low-quality wine were seized because they were sold as organic for a higher price ^1^.	[57]
Italy, 2020	PDO, PGI, and organic wines	Substitution Counterfeiting Mislabelling	Mislabelling Deceiving practice	Low quality wine	More than one million litres of wine were seized because they were wrongly labelled as PDO, PGI (Protected Geographical Indications), and organic ^1^.	[57]
Italy, 2020	Prosecco and pinot grigio	Substitution Counterfeiting Mislabelling	Mislabelling Deceiving practice	Low quality wine	A wine company produced more than 35 million bottles of wine by mixing low-quality wine with wine that fulfilled the legislative requirements and sold them as prosecco and pinot grigio ^1^.	[54]
Italy, 2020	Tuscany wine	Substitution Tampering Counterfeiting	Mislabelling Deceiving practice	Sicily wine	Common wine from Sicily was sold as a prestigious wine with Geographical Indication from Tuscany. The bottles were imported from Turkey, whereas labels, corks, wooden boxes, and papers were produced in Bulgaria. The falsified bottles were 70% cheaper than the original wine.	[58]
**Meat**						
United Kingdom, 2017	Halal lamb	Substitution, Mislabelling Misleading information (according to Manning & Soon (2014))	Mislabelling Deceiving practice	Non-halal turkey	DNA testing confirmed that four representatives of a meat company were selling non-halal turkey labelled as halal lamb between 2013 and 2014 ^1^.	[59]
Belgium, 2018	Farmed chicken	Substitution Mislabelling	Mislabelling Deceiving practice	Organic chicken	The company that provides most of the poultry sold by butchers in province of Antwerp was selling farmed poultry meat labelled as organic ^1^.	[46]
Belgium, 2019	Organic meat	Substitution Mislabelling	Mislabelling Deceiving practice	Conventionally produced meat	Meat conventionally produced in The Netherlands was labelled and sold as organic meat in Belgium and Germany ^1^.	[29]
Italy, 2019	Japanese Kobe beef	Substitution Mislabelling	Mislabelling Deceiving practice	Regular beef	Regular beef was labelled and sold as Japanese Kobe beef ^1^.	[34]
Italy, 2019	Parma and San Daniele hams	Mislabelling Substitution Counterfeit	Mislabelling Deceiving practice	Hams with higher moisture content	About 35% of the Parma and San Daniele hams are produced from traditionally used pigs crossed with faster growing races. This meat with higher moisture content does not fulfil the requirements needed for PDO label ^1^.	[43]
Spain, 2019	Iberian ham	Mislabelling Substitution Counterfeit	Mislabelling Deceiving practice	Other sort of ham	Iberian ham (*pata negra*) is produced from Iberian pigs fed with acorns during October–March. Taking into account the low acorn harvest in 2016/2017 that would be enough to feed 500,000 pigs, registration of over than 700,000 Iberian pigs pointed to probable food fraud ^1^.	[56]
**Other**						
Italy, 2019	Organic eggs	Mislabelling substitution	Mislabelling Deceiving practice	Farmed eggs	Eggs produced by hens kept in cages were labelled and sold as organic. To label eggs as organic, hens must spend at least one-third of their lives outdoors according to EU legislation ^1^.	[52]
Portugal, 2020	Organic eggs	Mislabelling substitution	Mislabelling Deceiving practice	Farmed eggs	Almost 50,000 farmed eggs were seized because they were labelled as produced by hens grown in open air ^1^.	[54]
Italy, 2019	PGI cheese	Mislabelling Substitution Counterfeit Misleading information (according to Manning & Soon (2014))	Mislabelling Deceiving practice	Other types of cheese	Three tons of cheese labelled as a Protected Geographical Indication (PGI) product without fulfilling the requirements were seized ^1^.	[31]
Italy, 2019	Pesto sauce	Mislabelling	Mislabelling Deceiving practice		A total of 600 kg of pesto sauce was found with falsified labels that masked the origin ^1^.	[31]
Italy, 2017	Buffalo milk	Mislabelling Substitution Counterfeit	Mislabelling Deceiving practice	Cow milk	Dairy companies used cow’s milk instead of buffalo’s milk, which is obligatory for the production of the Mozzarella di Bufala Campana (PDO) ^1^.	[59]
Italy, 2018	Tropea onion	Mislabelling Substitution Counterfeit	Mislabelling Deceiving practice	Onion	Twenty-three tons of onions labelled as Tropea onions (a sweet red onion variety protected by a PGI label) were seized. The onion was falsely labelled and actually produced in other regions ^1^.	[28]
Italy, 2017	Organic fruit and vegetables	Mislabelling Substitution	Mislabelling Deceiving practice	Conventionally produced fruit and vegetables	Fruits and vegetables were sold (in France, Germany, and UK) as organic despite being conventionally cultivated using pesticides. The fraud was estimated at one million euros since suspected farms received EU funds for organic production ^1^.	[27]
Italy, 2018	Several types of food	Mislabelling Substitution Counterfeit	Mislabelling Deceiving practice	Different origin	Eleven tons of different types of food were seized due to mislabelling. The products labelled as Prosciuto di Parma and Mozzarella di Bufala Campana had different origins, and some other products (tomatoes and different kinds of meat) lacked traceability documentation ^1^.	[28]
USA, 2018	Organic corn and soya	Mislabelling Substitution Misleading information	Mislabelling Deceiving practice	Non-organic corn and soybeans	Three US farmers were selling non-organic corn and soybeans as organic. The fraud remained undetected for eight years because the farmers could hire their own control bodies who did the testing that differentiated organic from conventional food in exceptional cases only ^1^.	[40]
Italy, 2018	Organic food	Mislabelling Misleading information	Mislabelling Deceiving practice	Non-organic food	Conventionally produced eggs, oranges, aromatic herbs, pasta, and fish were falsely labelled and sold as organic ^1^.	[60]
Italy, 2017	100% Arabica coffee	Mislabelling Substitution Misleading information	Mislabelling Deceiving practice	Non-Arabica coffee	A coffee reseller mixed Guatemalan coffee with other coffee from Vietnam and Uganda and labelled its product as 100% Arabica from Guatemala. The financial authorities seized 110,000 boxes of the suspected coffee with a total value of 500,000 EUR ^1^.	[53]
World, 2018	Coffee Arabica	Mislabelling Substitution Misleading information	Mislabelling Deceiving practice	Coffee Robusta	Analyses revealed that 10% of the coffee samples tested contained from 1.6% to more than 21% of coffee Robusta although they were labelled as the more expensive coffee Arabica ^1^.	[46]

* Unauthorized Food Manipulation (types of criminal offenses) according to Croatian Criminal Law (NN 125/11, 144/12, 56/15, 61/15, 101/17, 118/18, 126/19, 84/21): ^1^ Article 236: Fraud [9].

**Table 3:** Unauthorized Food Manipulation regarding contamination in food products, 2010–2020.

Country, Year	Food	Unauthorized Food Manipulation *		Adulterants	Case	Reference
		Fraud Type (USA): (a) FDA 401, 403 (b) Spink & Moyer (2011) (c) Manning & Soon (2016)	Fraud Type (EU) 178/02, article8			
**Honey**						
Europe, 2017	Honey combs	Adulteration Substitution	Mislabelling Deceiving practice	Paraffin, stearin	Beeswax intended for use as a base for honey combs was adulterated with paraffin and stearin in order to make a profit. Apart from risks for bee health related to the presence of stearin and paraffin in beeswax, consumers can eat these harmful adulterants incorporated in honey combs ^1,2^.	[61]
**Oil**						
China, 2011	Cooking oil	Adulteration Substitution	Mislabelling Deceiving practice	Recycled cooking oil	In China, potentially cancerogenic recycled cooking oil is often collected from sewage drains and gutters behind cooking facilities and then sold to restaurants ^1,2,3^.	[62]
Morocco, 2018	Olive oil	Adulteration Counterfeiting	Mislabelling Deceiving practice	Olive-oil-like capsules	Unknown toxic substances in capsules were mixed with water for the unauthorized production of olive oil and sold as olive oil in several regions of Morocco ^1,2,3^.	[48]
Brazil, 2020	Olive oil	Adulteration Tampering Substitution	Mislabelling Deceiving practice	Lampante olive oil	According to the Brazilian authorities, 64% of analysed samples of olive oil in the last two years were mislabelled. Some of the oils labelled as olive oil (15%) contained lampante olive oil (intended for use in lamps) that is not fit for human consumption ^1,2,3^.	[30]
Taipei, 2020	High quality olive oil	Adulteration Substitution	Mislabelling Deceiving practice	Low grade palm oil	A food-processing company was accused of falsely labelling low-grade palm oil and other cheap oils as high quality olive oil. These blends also contained artificial colorants that were harmful to human health ^1,2,3^.	[57]
**Alcoholic drinks**						
Czech Republic 2012	Becherovka and other liquors	Adulteration Counterfeiting Mislabelling	Mislabelling Deceiving practice	Methanol	Due to methanol poisoning, 41 people died and many more were admitted to hospital. The sources of the methanol were liquors, so the government banned the sale of liquors with more than 20% alcohol to prevent further health damage ^1,2,3^.	[63]
Cambodia, 2012	Rice wine	Adulteration Counterfeiting Mislabelling	Mislabelling Deceiving practice	Methanol	After drinking rice wine contaminated by methanol, 49 people died and more than 300 people were hospitalized ^1,2,3^.	[8]
Ukraine, 2016	Vodka	Mislabelling Adulteration	Mislabelling Deceiving practice	Methanol	Thirty-eight people died after drinking vodka made from methanol ^1,2,3^.	[64]
India, 2017	Alcohol	Adulteration Substitution	Deceiving practice	Methanol, antifreeze	At least twelve people died after consuming illegally produced alcohol that contained toxic substances (methanol and antifreeze agent) added to increase alcohol strength ^1,2,3^.	[65]
Italy, 2018	Wine	Adulteration Mislabelling	Mislabelling Deceiving practice	Synthetic aromas	A total of 3000 hectoliters of poor-quality synthetic wine aromas were added to deceive consumers ^1^.	[47]
Belgium, 2020	Red wine	Adulteration Counterfeiting Mislabelling	Mislabelling Deceiving practice	MDMA, MDA	A woman died after drinking red wine Merlot Cabernet Sauvignon 2016 that contained high levels of the amphetamine-like stimulants MDMA (ecstasy) and MDA. The counterfeited wine had a brown cork, while genuine wine (brand Black & Bianco) has a black cork ^1,2,3^.	[54]
Kuwait, 2020	Alcoholic drink	Adulteration Counterfeiting mislabelling Homicide	Mislabelling Deceiving practice Murder	Alcohol for perfume production	Four people died and six were in critical condition in Kuwait after drinking alcoholic beverages with alcohol intended for perfumes, not for producing alcoholic beverages ^1,2,3^.	[35]
Mexico and Dominican Republic, 2020	Alcoholic drink	Adulteration Counterfeiting Mislabelling Homicide	Mislabelling Deceiving practice Murder	Methanol	In Mexico and the Dominican Republic, 105 and 177 people, respectively, died after drinking fraudulently produced alcoholic drinks that contained methanol ^1,2,3^.	[66]
**Meat**						
Brazil, 2017	Fresh meat	Adulteration Dilution Mislabelling	Mislabelling Deceiving practice	Non-fresh meat, meat contaminated with Salmonella	A huge food fraud was discovered in the Brazilian meat sector. Several malpractices were carried out: intentional distribution of meat contaminated with Salmonella, adding chemicals to make meat look fresh, adding water to increase weight, adding soy to increase protein content. Brazil, the world’s largest exporter of beef and chicken, had exportation losses corresponding to 0.2% of its GDP ^1,2,3^.	[67]
Brazil, 2018	Chicken meat	Simulation (illegitimate product looks as legitimate)	Deceiving practice	Chicken meat contaminated with Salmonella	A few official control laboratories were accused of replacing samples of meat contaminated with Salmonella with meat samples that fulfilled the legislative criteria. In this way, the contaminated meat had health certificates required for export to the EU. The EU banned entries of the affected lots of frozen chicken meat ^1,2,3^.	[50]
Kenya, 2018	Fresh meat	Adulteration Mislabelling	Mislabelling Deceiving practice	Non-fresh meat	Some butchers treated meat with sodium metabisulfite that give meat a red color for weeks. This chemical can induce allergic reactions to consumers who are sensitive to sulfites ^1,2,3^.	[39]
Spain, 2018	Meat	Adulteration Tampering Mislabelling Dilution	Mislabelling Deceiving practice	Meat contaminated with Salmonella	Fifty tons of meat that posed a risk to human health were seized. The meat was intended to be sold to schools, restaurants, and hotels. Several illicit practices were discovered: mislabelling, defrosting of the meat by adding warm water, and the addition of viscera and pork blood to increase its weight. Some of the seized meat expired more than three years earlier and was contaminated with Salmonella ^1,2,3^.	[50]
Portugal, 2019	Fresh meat	Adulteration Mislabelling	Mislabelling Deceiving practice	Non-fresh meat	Many analysed meat samples contained sulphite, a forbidden substance added to meat to enhance appearance by inhibiting discoloration ^1,2^.	
**Eggs**						
Netherlands, 2016	Eggs	Adulteration Tampering Unauthorized chicken treatment	Mislabelling Deceiving practice	Eggs contaminated with fipronil	The presence of fipronil residues in eggs was probably caused by the illegal use of the chemical on farms in the Netherlands to control red mites in food-producing animals (chickens). This illegal activity has resulted in fipronil detected in eggs and chicken meat. The measured levels in some samples of eggs exceeded EU limits. If consumed in large quantities, fipronil is nephrotoxic and hepatotoxic ^1,2^.	[68]
Taiwan, 2018	Eggs	Adulteration Mislabelling Tampering	Mislabelling Deceiving practice	Expired contaminated eggs	Large distributors of eggs recalled its eggs because they contained antiprotozoal agent nicarbazin, which is forbidden in Taiwan. The company was accused for mislabelling the recalled eggs to extend expiry dates with the intention of selling to restaurants, hotels, and bakeries ^1,2^.	[40]
Austria, 2020	Eggs	Adulteration	Mislabelling Deceiving practice	Rotten eggs	A big distributor was accused of mixing into their products rotten eggs stored for several months, some of them contaminated with chicken feces ^1,2^.	[54]
**Sea food**						
Argentina, Brazil 2017	Shrimps	Adulteration Tampering	Mislabelling Deceiving practice	Sodium tripolyphosphate	Investigators seized 400 kg of the shrimps treated with sodium tripolyphosphate, a forbidden chemical used to retain water and artificially increase the weight of the product ^1,2^.	[38]
Europe, 2017	Fresh tuna	Adulteration Tampering	Mislabelling Deceiving practice	Non-fresh tuna nitrites/nitrates, carbon monoxide	During the EU-coordinated action Europol OPSON VII, it was discovered that tuna intended for canning was sold as fresh. Tuna was treated with chemical substances such as nitrites/nitrates, additives containing high level of nitrites, and/or carbon monoxide that altered its color to give the impression of its freshness. In total, more than 51 tons of tuna were seized and more than 380 samples were taken. Consequently, an increased number of scombroid poisonings (165 cases in 2017) was reported after the ingestion of tuna with high histamine levels due to poor quality. Additionally, the used nitrites may have led to formation of cancerogenic nitrosamines ^1,2,3^.	[69]
France, 2018	Fish (fresh tuna)	Adulteration Mislabelling	Mislabelling Deceiving practice	Salt, potassium lactate, potassium acetate, citric acid, polyphosphate, nitrates, nitrites, ascorbic acid	French authorities received a report that, apart from adding water to increase fish weight up to 30%, salt, potassium lactate, potassium acetate, citric acid, and polyphosphate were also added for retaining water. To give tuna a red color, carcinogenic nitrates and nitrites, as well as ascorbic acid were also added ^1,2^.	[70]
**Dairy products**						
India, 2017	Milk	Adulteration Dilution	Mislabelling Deceiving practice	Contaminated water, sodium hydroxide, detergents, starch, sugar, urea	According to the findings of Indian authorities, 30% of the milk sold in India is adulterated. Adding contaminated water to increase the volume can have implications for consumers’ health. Sodium hydroxide, detergents, starch, sugar, and urea have also been detected in the adulterated milk ^1,2^.	[70]
India, 2017	Milk	Adulteration Substitution	Deceiving practice	Glucose and detergents	The authorities discovered 1000 litres of the so-called dairy product “synthetic milk” that contained glucose and detergents ^1,2^.	[49]
Italy, 2017	Cheese	Adulteration Substitution	Mislabelling Deceiving practice	Spoiled milk treated with caustic soda	It was discovered that spoiled milk was treated with caustic soda in the production of Mozzarella di Bufala Campana. This chemical was used to mask acidification and aging ^1,2^.	[59]
Brazil, 2018	Milk powder	Mislabelling Adulteration	Mislabelling Deceiving practice	Sugars and other non-authorized substances	During the production of milk powder, sugars and other non-authorized substances were added ^1,2^.	[48]
India, 2019	Milk	Adulteration Mislabelling	Deceiving practice Mislabelling	Palm oil, detergent, and other chemicals	The Indian authorities have dismantled a unit producing fraudulent milk not fit for human consumption. The owners of the factory transformed 5000 litres of milk into 15,000–20,000 litres by adding substances such as palm oil, detergent, and other chemicals, which was then distributed in the area ^1,2^.	[52]
Pakistan, 2019	Milk	Adulteration Mislabelling	Mislabelling Deceiving practice	Detergent, shampoo, urea, washing powder, and formaldehyde	Some producers of milk in Pakistan sold milk adulterated with detergent, shampoo, urea, washing powder, and formaldehyde. Formaldehyde is carcinogenic and used as a preservative. Pakistan is the fifth largest producer of milk in the world ^1,2,3^.	[42]
Bangladesh, 2020	Milk	Adulteration Counterfeiting Mislabelling	Mislabelling Deceiving practice	Detergent, urea, synthetic milk powder	Milk adulterated with detergent powder, urea fertilizer and synthetic milk powder caused severe diseases affecting the kidneys, stomach, and intestine ^1,2,3^.	[71]
**Spices**						
India, 2018	Spices	Adulteration Dilution	Mislabelling Deceiving practice	Grass, rice husk, wheat, salt, dyes	Spices such as turmeric, chili powder, fennel, and coriander were mixed in spice production plants with adulterants like grass, rice husk, wheat, or salt. These substances were dyed with unauthorized colors and used to dilute the spices ^1,2^.	[41]
India, 2019	Spices	Adulteration Dilution Substitution	Mislabelling Deceiving practice	Wood dust, red brick powder, corn flour, sodium sulfoxylate	The company was selling adulterated spices: wood dust as coriander powder, red brick powder as red chili powder, corn flour as gram (chickpea) flour, and sodium sulfoxylate as jaggery (unrefined sugar made from sugar cane or palm) ^1,2,3^.	[29]
Pakistan, 2019	Spices	Adulteration	Mislabelling Deceiving practice	Rice husk and different colorants	Rice husk was added to chili powder, and different colorants were used to increase the color of the spices. In the same factory, 1700 kg of rice and 16 kg of colorants were seized ^1,2^.	[51]
Spain, 2019	Spices (saffron)	Adulteration Dilution Substitution	Mislabelling Deceiving practice	Plant extracts, chemical reagents	In a production plant, genuine saffron was mixed with parts of the plant not considered food, as well as with extracts from other plants and chemical reagents ^1,2^.	[56]
USA, 2019	Spices (curcuma)	Adulteration	Deceiving practice	Lead chromate	Stanford University detected lead chromate in curcuma that was produced in Bangladesh. Some samples contained more than 500 times the maximum lead amount allowed in US ^1,2,3^.	[52]
Pakistan, 2020	Spices	Adulteration Substitution Mislabelling	Mislabelling Deceiving practice	Nonfood-grade colorants	Chili powder was mixed with dangerous substances such as non-food-grade colorants and other chemicals ^1,2^.	[54]
**Other**						
Pakistan, 2019	Tea	Adulteration Mislabelling	Mislabelling Deceiving practice	Colors, bark	Authorities seized more than two tons of black tea adulterated with colors and bark ^1^.	[31]
Bangladesh, 2018	Fruit juice	Adulteration Substitution, Mislabelling	Mislabelling Deceiving practice	Chemicals	A factory produced fruit juice that did not contain any fruits and contained hazardous chemical substances ^1,2^.	[40]
Columbia, 2017	Sugar	Adulteration	Mislabelling Deceiving practice	Sulphur dioxide	About 850 kg of panela (unrefined whole cane sugar) was adulterated with sulphur dioxide to look fresher. The levels of adulterants were high enough to present a risk for consumers’ health ^1,2^.	[59]
India, 2017	Tea Sugar	Adulteration Mislabelling	Mislabelling Deceiving practice	Coal tar Sodium hydrosulphide	A batch of 1.5 tons of tea was adulterated with coloring agents extracted from coal tar to make the prepared tea to look stronger and more appealing. It makes the prepared tea appear stronger, and thus more easily sold. In the same city, 1620 kg of jaggery (solidified palm sugar) were adulterated with dyes (sodium hydrosulphide) to look more appealing ^1,2^.	[72]
Kenya, 2018	Brown sugar	Adulteration Mislabelling	Mislabelling Deceiving practice	Copper, mercury	Illegally imported Brazilian brown sugar was confiscated due to contaminations with copper and mercury, which are harmful to consumers. It was intended for transport to sugar factories in Kenya for further refining ^1,2,3^.	[60]
India, 2017	Snacks	Adulteration	Mislabelling Deceiving practice	Illegal dyes	Four hundred kg of snacks (e.g., chips, samosas, tomato sticks, Szechuan sticks) were confiscated due to the presence of illegal dyes ^1,2^.	[59]
Nigeria, 2016	Rice	Adulteration Substitution Mislabelling	Mislabelling Deceiving practice	Sweet potatoes, synthetic resin, fragrance	The plastic rice entered the national market. This product is made by mixing sweet potatoes and synthetic resin formed into “grains”, which are then sprayed with a fragrance to mimic the smell of Wuchang rice ^1,2,3^.	[33]
China, 2011	Bean sprouts	Adulteration Mislabelling	Mislabelling Deceiving practice	Sodium nitrite, urea, antibiotics, 6-benzyladenine	Bean sprouts were treated with banned food additives (sodium nitrite, urea, antibiotics, and a plant hormone, 6-benzyladenine) to speed up growth and make vegetables look shinier ^1,2^.	[73]
China, 2011	Chinese cabbage	Adulteration	Mislabelling Deceiving practice	Formaldehyde	Vegetable distributors were discovered spraying a carcinogenic formaldehyde solution on Chinese cabbage to keep the products fresh during long transport to faraway markets ^1,2,3^.	[74]
Lebanon, 2016	Pickled turnips	Adulteration	Mislabelling Deceiving practice	Rhodamine B	Rhodamine B, a dye not permitted in food, was added to pickled turnips to accelerate the coloring process and enhance/preserve the coloring. Consumers ingest unauthorized colors, which is potentially both genotoxic and carcinogenic ^1,2,3^.	[75]
Italy, 2019	Mushrooms (truffles)	Adulteration Substitution	Mislabelling Deceiving practice	Bismethylthiomethane	Fifty companies were accused of selling “al tartufo” processed food online, in which the truffles were replaced by the synthetic aroma compound bismethylthiomethane (truffle sulphide) ^1^.	[43]
Germany, 2016	Hazelnuts	Adulteration Substitution Mislabelling	Mislabelling Deceiving practice	Peanuts	Georgian food companies were accused of mixing peanuts with hazelnuts and selling it as hazelnut for economic gain. These low-quality nut products were exported to Germany, where consumers complained after allergic reactions ^1,2^.	[76]

* Unauthorized food manipulation (types of criminal offenses) according to Croatian Criminal Law (NN 125/11, 144/12, 56/15, 61/15, 101/17, 118/18, 126/19, 84/21): ^1^ Article 236: Fraud; ^2^ Article 188: Production and marketing of a product harmful to human health; ^3^ Article 215: Endangering the life and property with generally dangerous act or medium [9].

Regarding our data from 2010 to 2020, the most frequent UFMs are conducted in meat and meat products, alcoholic drinks, honey, and dairy products. Food fraud was the most common in meat and meat products, seafood, and honey; origin masking in alcohol drinks and meat and meat products; and contamination in alcohol drinks, dairy products, and spices (Figure 2).

Regarding our data from 2010 to 2020, the most frequent UFM in wine, meat and meat products, and honey was mislabelling. Cheaper ingredients were incorporated predominantly in meat and meat products, seafood, honey, olive oil, and dairy products, while non-edible ingredients were added in meat and meat products, dairy products, and spices. Dilution and weight-increasing were the predominant UFMs in seafood and dairy products (Figure 3).

Spink and Moyer [17] delved into all of the possible areas of food crime. Typical examples of what constitutes a food crime are adulteration of ingredients in the final food product that have been altered (e.g., melamine added to milk, high fructose or glucose syrup added to honey). Next is tampering, meaning legitimate food products and food packaging used in a false manner (e.g., changed expiration date, setting another false declaration). A step further in the wrong direction is the over-run of a legitimate food product made from agreed quantities of the products but with insufficient product declarations, as it could stem from the black market. Another is the theft of a legitimate food product that has been stolen and released back into the legal market. In addition, there is diversion, which is the sale or distribution of legitimate food products outside of targeted or agreed upon stores (e.g., the finished food product is redirected to a market where there is no retail support). Another is simulation, which is a non-legitimate copy of a real food product designed to resemble a food product as legitimately as possible (e.g., a popular food product that has not been manufactured in compliance with all food safety procedures). Finally, there is counterfeiting, which is a broader concept of food adulteration, as it involves the theft of intellectual property and combines all aspects of product counterfeiting and packaging replication (e.g., a copy of a popular food product that has not been manufactured in compliance with all food safety procedures).

Manning and Soon [3] added a few more concepts of food fraud, namely the following: misleading information, that is, use of words such as “natural” or “traditional,” or the use of images or package design that do not reflect the contents or production methods; incorrect packaging, that is, the use of oversized packaging; as well as hazardous and malicious poisoning, bioterrorism, or sabotage, which is intentional food poisoning with the aim of causing fear and provoking terror.

In the US, a powerful response to food crime incidents was made in 1992 through establishing the Office of Criminal Investigation (OCI) by the Special Prosecutor and the Ministry of the Interior. The OCI is responsible for investigating criminal activity and legal violations within the FDA’s jurisdiction (i.e., food, cosmetics, damaged medical equipment, mislabelling of products, manufacturing and sale of counterfeit/unapproved drugs, product substitution, product damage, healthcare fraud, fraud involving the use of unapproved drugs, crimes involving the national blood supply, crimes involving false clinical studies, cybercrimes involving products within the FDA’s jurisdiction). 

Any evidence of UFM or tampering or adulteration of a food product in the US must be reported to the OCI, while all of the received food fraud information has to be forwarded to the relevant OCI field office. On the other hand, the Croatian Ministry of the Interior to this day does not have a similar unit or office to find and collect evidence and prosecute food crimes. Establishing a food crime office should be based on good experiences from the US, coupled with modern laboratory equipment and analytical methods including highly specialized technical bodies in the field of veterinary, food technology, agronomy, and sanitary engineering with practical experience. Other possibilities include close cooperation of certified and official public health or veterinary institutes with accredited (ISO 17025) analytical methods to detect food fraud (Figure 2). The best method for preventing food criminal manipulation is to improve the overall traceability of foods [19], as well as the implementation of a food defence system through food quality systems (FSSC 22,000 [77], BRC Food Standard 8 [78], PAS 96 [79], IFS Food Version 6.1 [80], SQF Quality Code [81], and Reg. 178/2002/EC [1]).

## 4. Laboratory Techniques for Food Fraud Detection

The development and implementation of food authentication is strenuous multidisciplinary work involving analytical methods combined with informatics, mathematics, and statistics [10].

The large number of substances that can be used for food fraud and the inability to evaluate them make conventional analytical methods inadequate for this purpose. New highly sensitive methods are capable of detecting illicit substances even if they are present only in trace amounts. The development of such methods is moving in the direction of implementing faster and more reliable analytical methods. The specificity of the methods is one of the basic requirements. The melamine incident, for example, occurred because the analytical method for determining protein content was not specific enough to distinguish protein from non-protein nitrogen [82].

The US Pharmacopeia (2012) favours an approach that tests food ingredients for authenticity rather than determining the absence of specific patterns. Well-known qualitative methods used for routine laboratory tasks are currently on the rise and are attracting greater interest, primarily because of their screening potential. 

Qualitative methods can be classified using a variety of criteria, but in all cases, they are used for questions that require binary responses (e.g., yes/no answers). When the answer is obtained from multiple non-specific signals, a multivariate classification strategy is required, also known as non-targeted screening [83]. This resource provides an overview of multivariate qualitative methods for food fraud detection, with examples and samples of adulterated foods. Some state-of-the-art methods and techniques, such as isotope-ratio mass spectrometry (IRMS), stable isotope analysis, genomics, and proteomics, should definitely be included in the methods to successfully confirm/or reject food authenticity. The application of these analytical methods provides insight into the accuracy and veracity of information about a product, primarily considering its chemical composition, origin, or production technology [10]. 

To reach the right conclusions, it is often necessary to interpret the results of the analysis using statistical (chemometric) methods [84]. Occasionally, problems can only be solved by using additional instrumental techniques that provide complementary information. Data fusion is an approach to obtain a single result from more than one source. There are three types of data fusion: low-level, mid-level, and high-level data fusion. The basis of each of these types is described in the literature [85,86], while Callao and Ruisánchez [83] summarized a set of studies on data fusion strategies in some food and quality-control processes. 

Hong et al. [87] highlighted some important food categories with a high incidence of adulteration in their literature search. The food categories were grouped into six: ‘animal origin and seafood’, ‘oils and fats’, ‘beverages’, ‘spices and sweeteners’, ‘grain-based food’, and ‘others’ for organic food and dietary supplements. Mass spectrometry (MS) is the most frequently used method in the analysis of spices and grain-based food. Liquid chromatography (LC) and gas chromatography (GC) are also frequently used for spices, oils, and organic foods. Nuclear magnetic resonance (NMR) is used frequently to discriminate the authenticity of oils, cereals, grains, and beverages. PCR is the predominant detection technology used for the analysis of meat and meat products, fish and seafood, and milk and milk products. Most applications addressed authentication issues and, to a lesser extent, adulteration. The analytical methods used to detect food frauds are categorized in the next sections.

### 4.1. Chromatographic Methods 

Frequently used techniques for food fraud detection include chromatographic analysis. Examples are thin-layer chromatography (TLC; e.g., high-performance liquid chromatography, HPLC; and GC) and MS-based methods (e.g., LC-MS/MS, GC/MSD) [87].

These methods allow rapid and reliable separation of similar chemical substances and, together, are considered one of the most important methods for food analysis. The methodology is based on the principle of establishing a unique chemical profile, making it possible to determine differences in the chemical composition of samples or to identify specific markers. Gas chromatography is used to analyse volatile compounds, while liquid chromatography is used to evaluate carbohydrates, proteins, vitamins, phenolic compounds, amino acids, and pigments. By combining these chromatographic methods with mass spectrometry, it is possible to obtain structural information that cannot be obtained by other analyses. Chromatographic methods are most commonly used in the analysis of honey, wine, oil, coffee, milk, cheese and mushrooms to determine fatty acids, triglycerides, sterols, hydrocarbons, tocopherols, and alcohol, as these compounds define the specific chemical profile of the product [10]. Other applications with HPLC and LC techniques are used for fruits, fruit juices, and sweeteners, while LC and GC are also used to a significant degree for spices, oils, and organic foods [87].

Mass spectrometry (MS) accounted for the largest proportion at 20.6%, PCR for 18.5%, and LC for 11.6%. It is interesting how MS is used extensively in most food categories, being also the most frequently used method in the analysis of spices, extracts, cereals, grains, and pulses, as well as accounting for the greatest proportion in Asian countries and South Korea (20.7% and 38.1%, respectively) [87].

### 4.2. Spectroscopic Analysis

Infrared spectroscopy is a fast and non-destructive method by which functional groups in a molecule can be identified. It operates in the range of electromagnetic radiation of 2.5–15.0 μm and is based on the absorption of light whose frequency coincides with the vibration frequency of the bonds in a molecule. Nuclear magnetic spectroscopy works on the principle of absorbing the energy of the nucleus in the field of radio waves. These methods allow the collection of spectroscopic and structural information, which is then used to verify the authenticity of the product [88]. They are used, for example, to detect melamine in milk and identify soy protein in meat products. Other authors described how NMR is frequently used to discriminate the authenticity of oils, cereals, grains, alcoholic beverages, and fruit juices [87].

### 4.3. Stable Isotope Analysis 

One of the most promising and popular state-of-the-art techniques for the protection and discrimination of geographical origin (the Protected Designation of Origin (PDO)) and avoidance of fraudulent labelling of milk and dairy products has been using multi-element and multi-isotope-ratio analysis with isotope-ratio mass spectrometry (IRMS), inductively coupled plasma mass spectrometry (ICP-MS), and ICP-AES [89,90]. Some important results have demonstrated that stable isotope ratios of C, N, O, S, and Sr of milk and cheese were linked to their territories of origin, as a result of variations in the type of vegetation and the environment [91].

Improved detection of water addition in fruit juices was developed by conducting an analysis of the isotopic ratios of oxygen (18O/16O) of ethanol derived from the sugars in orange juice using the preparation steps of the SNIF-NMR method followed by IRMS [92].

The geographical origin of premium long-grain rice can be analysed using IRMS, but also using ICP-MS [93]. 

For identifying the addition of low-cost commercial sugar syrups (beet and cane syrup) to pure apple juices and related products, an improved procedure for determining 13C and 2H isotope ratios using gas chromatography-isotope ratio mass spectrometry (GC-IRMS) has been developed [94]. 

Hydrogen and oxygen (originating from water used by animals and plants) have light and heavy isotopes, and their ratio is a unique marker of the climatic and geographical area. Carbon and nitrogen isotopes can be used to distinguish plants that feed animals, and therefore their geographical origin can be determined. The design of isotopic maps of Europe is underway so that the authenticity of regional products (e.g., champagne and Scottish salmon) can be verified more easily [95].

### 4.4. Molecular Methods

Restriction fragment length polymorphism polymerase chain reaction (PCR-RFLP) and real-time single or multiple polymerase chain reaction (RT-PCR) are based on DNA fragment amplification and hybridization with specific assays to identify different origins of meats in the samples. DNA barcoding (DNA sequencing using a validated protocol) is a powerful technique in the analysis of meat and fish products, as long as the DNA is preserved in the samples. It is effective in determining the origins of raw products and detecting adulterated foods (e.g., mixing products of different taxonomies such as rice and ginseng). Recently, the method has been advanced to next-generation sequencing and DNA-meta-barcoding, so it is possible to identify meat types in the mixtures [88]. Some authors determined PCR as the predominant detection technology used for the food categories fish and seafood, and milk and milk products [87].

### 4.5. Immunological Methods

These methods are based on the reactions between the antibodies and the antigens. The use of these methods is quite widespread given that they are fast, sensitive, highly specific, and easy to perform. Currently, the most common method for the analysis of meat, dairy, and fish products is the enzyme-linked immunosorbent assay (ELISA). 

Since the detection and discrimination of cereal contamination in gluten-free foods is essential for celiac disease patients, some ELISA methods have been established for the specific discrimination of wheat, rye, barley, and oats in gluten-free food (apart from more expensive and less user-friendly methods with real-time PCR and quantitative competitive (QC)-PCR) [96,97]. Recently, food fraud in cereal food products derived from a ready-to-eat powder (e.g., Sunsik) has been a great concern for regulatory authorities and consumers, especially the mislabelling or incorrect labelling of allergenic constituents in Korea. Thus, specific primers for each species have been developed based on sequence polymorphisms in chloroplast rpoC2m, and multiplex PCR can detect components of commercial flow-mixed products [98].

IR spectroscopy, Raman, immunosorbent assays (e.g., ELISA), and biosensors are used less compared to the other detection methods for food authenticity [87].

## 5. Preventive Measures

Food safety hazards are defined as “*biological, chemical or physical hazards or a food condition with the potential to cause adverse health effects*” [3,99]. Food hazards can be physical (e.g., glass, metal, foreign objects), biological (e.g., bacteria or parasites), and chemical (e.g., unapproved additives, banned chemicals, antibiotics, pesticides). 

In food processing, introducing fraudulent raw materials into food (e.g., through adulteration or counterfeiting or intellectual property theft), or to use those raw materials in an inferior quality, affects the product itself. In addition, in poorly controlled production facilities, especially with institutionalized food (e.g., hotels, restaurants, nursing homes), falsifying packaging (e.g., through tampering or simulation) during distribution or prior to purchase of a final food product can occur [100]. Dilution of the product with water, mixing it with cheaper raw materials, or using illicit additives and chemical agents to improve sensory properties can occur at any point in the food supply chain. Food products may contain residues of pesticides and antibiotics, exceeding MRL levels at any part of the production or at any stage of the food chain (from farm to fork).

The widespread and fragmented food supply chain of today is increasingly vulnerable to fraud, which can occur anytime and in every part of food supply chain [101]. Since food production ingredients nowadays come from all over the world, food fraud could happen in one country if fraudulent ingredients were added in another country. Food ingredients can be adulterated at any stage of the food chain, whether they are the basic ingredient or merely raw material. They can occur during cultivation, harvest, slaughter, processing, storage, or transportation. Therefore, to prevent malicious food contamination, criminal and UFM, food protection principles should be considered in every part of the supply chain or processing [102]. 

The answer to preventing food fraud with ingredients required for the food’s production is in quality management (QM) that contains food defence systems (FSCC 22000, BRC Food Standard 8, PAS 96, IFS Food Version 6.1, SQF Quality Code) in FBO facilities, as well as food defence certification on packaging or during the determination of traceability. Food safety guidance has also been issued by the FDA to all food manufacturers, food processors, and transporters, importers, bottlers, grocery stores, and restaurants, as well as dairy and cosmetic processors and transporters [103].

### Food Fraud Preventive Activities

Food defence is the answer to protecting products from UFM (Figure 1). To assess the risk/benefit ratio of the food system in relation to the occurrence of criminal events, the Threat Assessment of Critical Control Points (TACCP) and the Vulnerability Assessment of Critical Control Points (VACCP) are used. 

There are three basic elements that need to be considered when protecting the food chain, namely event prevention, event response, and event recovery [104]. In terms of preventive measures, the food business management structure should develop a preventive strategy for the possibility of malicious food contamination or UFM, facility control measures, product withdrawal procedures, investigation of suspicious activities, and evaluation of implemented procedures. Vulnerability assessment should be conducted through critical control points (VACCP) of the food-processing facility and the entire supply chain before a food defence system is established [105].

Points in the food processing facility that contain specific stages where ingredients are added and mixed into a food product are often identified as high risk, as is accessibility to open ingredients. Similarly, points where liquid ingredients are handled or stored are often identified as high risk. Care must be taken in the design of risk mitigation strategies at these important stages [100]. 

To assess vulnerability and rank critical issues, the FDA has provided an application that calculates the CAV (Criticality + Accessibility + Vulnerability) score, which is the shorter version of the CARVER software. This simplifies and standardizes vulnerability assessments and points out exposure and potential hazards by making the investigation simple and independent to investigate. The program allows quantification of vulnerabilities for each of the characteristics. It also examines the economic, psychological, and public health consequences of potential food attacks [106]. The vulnerability analysis is carried out differently depending on whether the possible origin of the fraud is the raw materials, packaging material, processing at FBO, or in their distribution. When assessing food product vulnerabilities, a few factors have to be taken into account: history of product fraud incidents, economic factors, ease of fraudulent activity, supply chain complexity, current control measures, and supplier confidence [107,108].

The mitigation strategy is a practice that FBOs must implement with the goal of significantly reducing or eliminating the critical control points previously identified in the vulnerability assessment. It can be implemented throughout the food production process, farming and livestock, through food processing, distribution, storage, and retail. The general and focused mitigation strategy protects against unwanted criminal food manipulation or intentional food contamination. There is software available for developing a mitigation strategy for consequences of an attack on the food supply chain, which was also developed by the FDA.

## 6. Response to Food Accidents and Difficulties in Controlling Fraud

**Figure 4:**
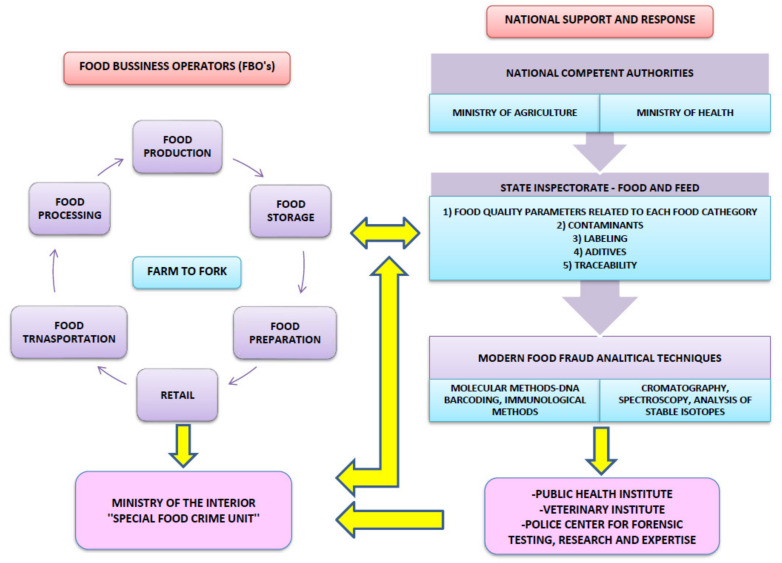
Responding to food fraud activities (Croatian system).

The response plan should contain contact information (the police, the state inspectorate, ministry of agriculture, ministry for health) in case of a fraudulent incident or intentional contamination in the food supply chain (Figure 4). 

The left part of the picture shows some of the most important steps within which every FBO should internally ensure high-quality traceability, to later facilitate official supervisors’ insight into the business, from raw material procurement to the marketing of the finished product. The left picture reveals a complex institutional structure that needs to provide effective support as well as inspection of FBOs and food in warehouses and retail. It is evident that the key to preventing food fraud in the market is good cooperation and well-coordinated activities between all official institutions that need to communicate frequently and effectively. It is necessary to employ state-of-the-art analytical techniques to ensure adequate completion of official controls, as well as adequate sampling that must be representative and sufficiently frequent. Considering that it is most important that there is no food on the market that could endanger the health of consumers, methods for the presence of food contaminants should be implemented: for example, hidden pharmacological substances, polycyclic aromatic hydrocarbons (PAHs), dioxins, and polychlorinated biphenyls (PCBs). The food quality parameters related to each food category, on the other side, should detect deceptions that are not dangerous to the health of the consumer but that diminish its nutritional value (e.g., by replacing olive oil with cheaper substitutions by adding chlorophyll, adding water or apple juice to red berry juices, sugar syrups to honey, etc.). In order to successfully detect these food frauds, official laboratories need to have data from the sampling site, but more than that, modern analytical techniques, applicable for food fraud detection. Unfortunately, many of them are very expensive and require special knowledge and education, while some require the creation of their own databases, which is a very time consuming and expensive process (e.g., isotope ratio mass spectrometry; IRMS).

State institutions should have procedures for food emergency cases. For instance, Article 19 within the EU General Food Law (Reg (EC) 178/2002) [1] listed all responsibilities for FBOs, and when food should be withdrawn from the market. Some of the most important highlights are set in several bullet-points, such as:

*“1. If a FBO considers or has reason to believe that a food which it has imported, produced, processed, manufactured or distributed is not in compliance with the food safety requirements, it shall immediately initiate procedures to withdraw the food in question from the market where the food has left the immediate control of that initial food business operator and inform the competent authorities thereof. Where the product may have reached the consumer, the operator shall effectively and accurately inform the consumers of the reason for its withdrawal, and if necessary, recall from consumers products already supplied to them when other measures are not sufficient to achieve a high level of health protection.*

*2. FBOs responsible for retail or distribution activities which do not affect the packaging, labelling, safety or integrity of the food shall, within the limits of its respective activities, initiate procedures to withdraw from the market products not in compliance with the food-safety requirements and shall participate in contributing to the safety of the food by passing on relevant information necessary to trace a food, cooperating in the action taken by producers, processors, manufacturers and/or the competent authorities.*

*3. The FBO shall immediately inform the competent authorities if it considers or has reason to believe that a food which it has placed on the market may be injurious to human health. Operators shall inform the competent authorities of the action taken to prevent risks to the final consumer and shall not prevent or discourage any person from cooperating, in accordance with national law and legal practice, with the competent authorities, where this may prevent, reduce or eliminate a risk arising from a food.*

*4. The FBO shall collaborate with the competent authorities on action taken to avoid or reduce risks posed by a food which they supply or have supplied (Reg (EC) 178/2002).”*

After the incident, FBOs should restart food manufacturing and gain consumers’ confidence that their products are not adulterated, which is a very demanding process, but still possible with time. The question is whether this will be profitable after economic losses and possible human casualties (major poisonings). Therefore, the food system recoverability (time for system recovery) could be scored in the food defence plan evaluation in this criteria: >1 year, 9–10; 6 months to 1 year, 7–8; 3–6 months, 5–6; 1–3 months, 3–4; <1 month, 1–2 [106].

## 7. Conclusions

Food fraud is an increasingly problematic phenomenon, whose presence may be significantly reduced by FBOs, by implementing a food quality management system and food defence principles within production (FSCC 22000, BRC Food Standard 8, PAS 96, IFS Food Version 6.1, SQF Quality Code), in order for manufacturers that have an interest to retain the quality of the final food product, as well as consumers’ trust. Respectable food industries have already implemented such food quality systems, and the final products are made from certified ingredients. However, less considerate parties inside the food chain could have different aims, as their goal might be to use cheaper components, so they change products in their most important attributes. Hence, they might lie about the ingredients/composition on packaging or give false and misleading statements on labels. The general purpose of such behaviour is to gain economic profit, which is a necessary component of food fraud, and food crime as well. Finally, such violations of food law are criminal offenses that must be prosecuted by the police and the court. Police work involves gathering material evidence and prosecuting perpetrators in front of the court, but the police office for food fraud should have knowledge of food safety, processing, and production. 

The possible subsequent improvements regarding food fraud prevention could be education and development of awareness of potential food crime that could be committed with UFM, as well as more extensive institutional monitoring and penalisation. 

Any evidence of UFM or tampering or adulteration of a food product in the US must be reported to the OCI, while all of the received food fraud information has to be forwarded to the competent OCI field office. On the other hand, the Croatian Ministry of the Interior to this day does not have a similar unit or office to find and collect evidence and prosecute food crimes. Establishing the Food Crime Office should be based on good experiences from the US, coupled with modern laboratory equipment and analytical methods including highly specialized technical bodies in the field of veterinary, food technology, agronomy, and sanitary engineering with practical experience. Other possibilities include close cooperation with certified and official public health or veterinary institutes with accredited (ISO 17025) analytical methods to detect food fraud.

Possible challenges are expensive and demanding analytical techniques, analysing large datasets, and perhaps a lack of food-related regulations, which should be addressed in the near future. Intentional adulteration or UFM could cause serious consequences to public health or economy. Therefore, the general public could lose confidence in food safety at the global level, but also in the government’s ability to protect them. The solution is in increasing the levels of risk-based inspections, monitoring and implementation of food defence systems throughout the entire food supply chain, and better control of ingredients and their certifications. Prevention of fraud in the food chain and promotion of authentic food products are undoubtedly key elements in the successful placement of food products on the world market.
